# P45 Translating AWaRe-based antibiotic use quality indicators into practice: an automated framework and dashboard leveraging Global-PPS data

**DOI:** 10.1093/jacamr/dlaf230.052

**Published:** 2025-12-04

**Authors:** Hossam Almadhoon, Baboucarr Njie, Annie Heath, Annelies Boven, Ines Pauwels, Juliet Namugambe, Ann Versporten, Erika Vlieghe, Michael Sharland, Aislinn Cook

**Affiliations:** Centre for Neonatal and Paediatric Infection, Institute of Infection and Immunity, School of Health & Medical Sciences, City St George’s, University of London, London, UK; Centre for Neonatal and Paediatric Infection, Institute of Infection and Immunity, School of Health & Medical Sciences, City St George’s, University of London, London, UK; Centre for Neonatal and Paediatric Infection, Institute of Infection and Immunity, School of Health & Medical Sciences, City St George’s, University of London, London, UK; Global Health Institute, University of Antwerp, Antwerp, Belgium; Global Health Institute, University of Antwerp, Antwerp, Belgium; Centre for Neonatal and Paediatric Infection, Institute of Infection and Immunity, School of Health & Medical Sciences, City St George’s, University of London, London, UK; Global Health Institute, University of Antwerp, Antwerp, Belgium; Global Health Institute, University of Antwerp, Antwerp, Belgium; Department of General Internal Medicine, Infectious Diseases and Tropical Medicine, University Hospital Antwerp, Antwerp, Belgium; Centre for Neonatal and Paediatric Infection, Institute of Infection and Immunity, School of Health & Medical Sciences, City St George’s, University of London, London, UK; Centre for Neonatal and Paediatric Infection, Institute of Infection and Immunity, School of Health & Medical Sciences, City St George’s, University of London, London, UK; Nuffield Department of Primary Care Health Sciences, University of Oxford, Oxford, UK

## Abstract

**Background:**

The WHO AWaRe Antibiotic Book provides concise, evidence-based guidance for antibiotic choice, dose and duration across more than 30 clinical infections in primary care and hospital settings. Building on this guidance, an international consensus process refined a set of 118 AWaRe-based quality indicators (QIs) to assess prescribing quality in these settings. While Global-PPS has reported real-time antimicrobial prescribing practices using the AWaRe classification since 2019, linking the AWaRe Book guidance to Global-PPS data has not yet been explored.

**Objectives:**

To develop an analytics framework and R Shiny dashboard that computes AWaRe QIs based on condition-specific Global-PPS prescribing data according to the AWaRe Book recommendations in inpatient and outpatient settings.

**Methods:**

AWaRe QIs measurable within Global-PPS, using diagnostic/indication codes for inpatients and symptom fields as proxy diagnoses for outpatients, were retained. These cover empirical treatment of common community-acquired infections and surgical prophylaxis; in this analysis, application was initially limited to adult prescriptions. Each QI measures specific prescribing dimensions: whether patients received oral (outpatient) or IV (inpatient) antibiotics; whether the agent was Access or Watch; and concordance with AWaRe recommended drug choice, dose and duration. For each included condition, AWaRe Book treatment recommendations were compiled into a structured reference table and mapped to Global-PPS variables capturing the corresponding fields (diagnosis/symptom codes, antibiotic, route, dose and frequency, prescribed duration (outpatient) and patient demographics). Algorithms written in R (RStudio) deterministically link structured Global-PPS Excel exports to the reference table, apply QI definitions and eligibility criteria and calculate numerators and denominators. Outputs are aggregated by ward/specialty and facility levels. The analytic pipeline was implemented and validated in R Markdown before being embedded in an R Shiny dashboard giving condition-specific tables and visualizations.

**Results:**

Thirty-eight AWaRe QIs were compatible with Global-PPS: 17 for inpatient care (covering sepsis, meningitis, pneumonia, intra-abdominal infections, upper urinary tract infections, complicated skin/soft tissue and osteoarticular infections and surgical prophylaxis) and 21 for outpatient care (respiratory tract, ear/nose/throat, dental, diarrhoeal, lymph-node, mild skin/soft tissue and lower urinary tract infections). Reports include case eligibility checks, hospital antimicrobial use profiles and QI-specific evaluations. The R Markdown prototype was piloted with retrospective Global-PPS datasets from inpatient and outpatient facilities in Africa and Asia. Discussions with Fleming Fund country partners highlighted the value of reusing existing PPS data to measure QIs and of condition-specific outputs that reveal deviations from WHO recommendations as potential stewardship targets. The dashboard processes de-identified Global-PPS exports uploaded by sites for temporary, session-based analysis; neither datasets nor outputs are stored or shared externally. Guidance will be provided for hospitals using other PPS methodologies (e.g. WHO-PPS) to adapt their data structure for use with the dashboard.

**Conclusions:**

Based on the AWaRe Antibiotic Book, this new framework generated automated, condition-specific quality assessments of antibiotic prescribing in both inpatient and outpatient settings. It provides guidance for stewardship priorities and enables tracking progress. Future work will extend to paediatrics and deepen integration with the Global-PPS system to strengthen antimicrobial stewardship globally.

